# TRANSITIONS OF ASSET POVERTY AND DEPRESSIVE SYMPTOMS: A COMPARISON BETWEEN URBAN AND RURAL CHINESE OLDER ADULTS

**DOI:** 10.1093/geroni/igae098.2508

**Published:** 2024-12-31

**Authors:** Ting Hu, Yu-Chih Chen

**Affiliations:** University of Illinois Urbana-Champaign, United States; The University of Hong Kong, Hong Kong, China (People’s Republic)

## Abstract

Urban-rural disparities and asset poverty are being examined individually as critical socioeconomic factors influencing depression among older adults. However, while asset poverty, a dynamic condition evolving over the life span, is significantly influenced by urban-rural residency and household registration (hukou) status in China, the extent of their contribution to the relationship between asset poverty transitions and depressive symptoms in later life remains largely unexplored. Using four waves of the China Health and Retirement Longitudinal Study, we examined how asset poverty affects depressive symptoms among 7,982 adults aged 60 and older and the interaction effects by rural/urban areas. The fixed effect regression models revealed that older adults who transitioned from a non-asset-poor status to an asset-poor status reported elevated levels of depression compared to those who maintained a non-asset-poor status. Furthermore, significant interaction effects were found in the relationship between asset poverty and depressive symptoms. The simple slope analyses revealed that the effect of transitioning to asset poverty on depression was only significant for older adults living in rural areas or with rural hukou. The findings highlight the necessity of prioritizing policies to build a financial support system for older adults and promoting asset building to mitigate the negative impacts of asset poverty on mental well-being in later life, particularly for those in rural areas or with rural hukou. Additionally, efforts at reducing urban-rural disparities in social and family support and within the social welfare system should be advocated.

